# Targeting malaria parasites inside mosquitoes: ecoevolutionary consequences

**DOI:** 10.1016/j.pt.2022.09.004

**Published:** 2022-10-05

**Authors:** Tsukushi Kamiya, Douglas G. Paton, Flaminia Catteruccia, Sarah E. Reece

**Affiliations:** 1 Centre for Interdisciplinary Research in Biology, Collège de France, Paris, France; 2 HRB Clinical Research Facility, National University of Ireland, Galway, Ireland; 3 Institute of Ecology and Evolution, and Institute of Immunology and Infection Research, School of Biological Sciences, University of Edinburgh, Edinburgh, UK; 4 Department of Immunology and Infectious Disease, Harvard T. H. Chan School of Public Health, Harvard University, Boston, MA, USA; 5 Howard Hughes Medical Institute, Boston, MA, USA

## Abstract

Proof-of-concept studies demonstrate that antimalarial drugs designed for human treatment can also be applied to mosquitoes to interrupt malaria transmission. Deploying a new control tool is ideally undertaken within a stewardship programme that maximises a drug’s lifespan by minimising the risk of resistance evolution and slowing its spread once emerged. We ask: what are the epidemiological and evolutionary consequences of targeting parasites within mosquitoes? Our synthesis argues that targeting parasites inside mosquitoes (i) can be modelled by readily expanding existing epidemiological frameworks; (ii) provides a functionally novel control method that has potential to be more robust to resistance evolution than targeting parasites in humans; and (iii) could extend the lifespan and clinical benefit of antimalarials used exclusively to treat humans.

## Preventing transmission by targeting parasites within mosquitoes

Malaria is an infectious disease caused by apicomplexan parasites of the genus *Plasmodium* and is transmitted through the bite of an insect vector, most commonly mosquitoes of the genus *Anopheles. Plasmodium* parasites – *P. falciparum* chief among them – cause over 240 million infections in humans each year and are responsible for over half a million annual deaths globally [1]. A wide array of malaria-control and elimination strategies – including insecticides, bed nets, antimalarial chemotherapy, surveillance and diagnostics – have contributed to a large reduction in the global malaria burden over the past few decades, and a partially effective vaccine is available. However, counter-evolution of the targeted organisms (i.e., mosquitoes resist insecticides, and parasites resist antimalarial drugs) threatens the future of malaria control [2].

The evolution of resistance to insecticides takes many forms, including alterations to the mosquito’s cuticle to reduce insecticide penetration [3] and elevated metabolic detoxification [4]. Both mechanisms provide parasites with viable vectors and could reduce any cytotoxic effects of insecticides on parasite development [5]. When applied to humans, antimalarial chemotherapy targets blood-stage parasites, and **drug resistance/tolerance** (see Glossary) has evolved against nearly every antimalarial compound widely deployed, including the current frontline therapy, artemisinin derivatives [6], making novel interventions to augment the existing tool kit urgently needed.

**Figure 1.**
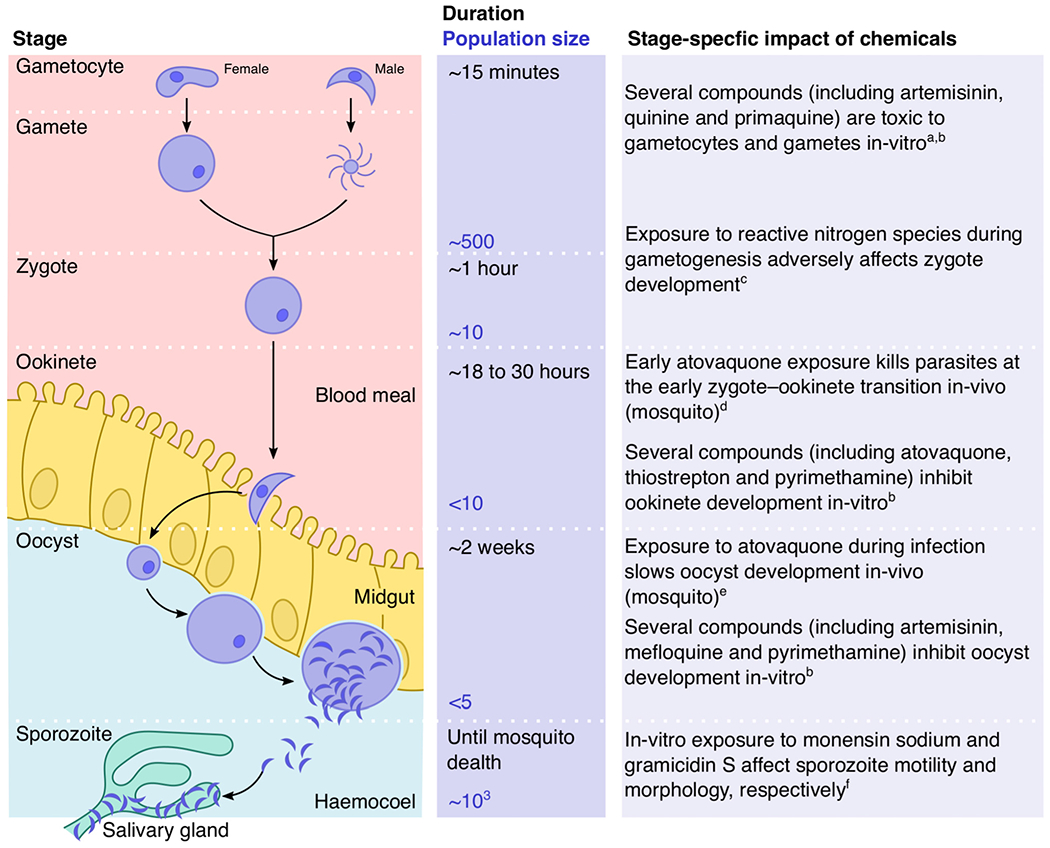
Adverse impacts of chemical compounds during *Plasmodium* sporogony. Parasites experience a series of population bottlenecks as they migrate around mosquitoes and transition between developmental stages. Each stage during sporogony presents a potential target for chemical intervention to reduce parasite transmission from mosquitoes. The stage duration and population sizes were adopted from [8]. Listed are non-exhaustive examples of adverse effects of chemical compounds at each stage of sporogony. Further examples are listed by [7,20]. The following superscripts refer to corresponding references: a, [20]; b, [7]; c, [79]; d, [21]; e, [14]; f, [80].

A promising new approach is to target malaria parasites inside mosquitoes (i.e., during **sporogony**) [7,8]. Here, we consider that sporogony spans from the point of **gametocyte** ingestion in a blood meal to when **sporozoites** exit the mosquito’s salivary glands upon entering a new mammalian host [9]. Sporogony involves a journey through several mosquito tissues and organs (Figure 1). Following a rapid sequence of gametocyte activation to form gametes, and fertilisation that gives rise to zygotes, parasites develop into motile **ookinetes** that penetrate the midgut epithelium. Once between the midgut epithelium and basal lamina, ookinetes differentiate into **oocysts**, which grow in size for 1–2 weeks as they undergo cell division to produce sporozoites. Once mature, each oocyst releases thousands of sporozoites into the haemolymph, and the mosquito becomes infectious when sporozoites reach the salivary glands. The transitions from gametocytes to ookinetes, and then to oocysts, present cascading bottlenecks for the parasite (Figure 1), making sporogony one of the most vulnerable stages of the parasite’s entire malaria life cycle [8,10].

Many chemical compounds are effective against *Plasmodium* parasites during sporogony (Figure 1). Efficacy has been demonstrated in several ways, including comparing the success of gametocytes and oocysts in mosquitoes following blood-feeding on treated humans [1–13], and giving already-infected mosquitoes a sugar feed containing antimalarials [14–16]. Every major class of antimalarials, as well as novel compounds, are efficacious in both *in vitro* and *in vivo* settings, building a consensus that parasites undergoing sporogony are plausible targets for chemical intervention (e.g., [7,17–19]; reviewed in [20]). More recently, Paton *et al.* demonstrated that tarsal exposure to atovaquone (i.e., via the mosquito’s legs) prevents the development of most ookinetes [21] and slows the development of surviving oocysts [14], blocking transmission in a dose-dependent fashion. Furthermore, atovaquone is equally effective against parasites in insecticide-resistant and -sensitive mosquitoes, highlighting the potential for reducing transmission even in areas with a high incidence of insecticide resistance [14,21]. Sporogony-targeting compounds can be delivered in multiple ways, including via bed nets, indoor sprays, eaves tubes, and sugar baits.

Malaria parasites have evolved resistance against almost every antimalarial deployed to treat humans; thus, it would be naïve to assume that compounds targeting parasites during sporogony will not be met with resistance evolution. Here, we consider the epidemiological and evolutionary consequences of chemically targeting parasites during sporogony. First, we demonstrate that existing epidemiological models can be readily extended to describe how targeting parasites in the mosquito vector affects parasite fitness. Second, we consider evolutionary outcomes, revealing why targeting parasites during sporogony may produce different evolutionary outcomes compared to using antimalarial therapy only for humans or using insecticides against mosquitoes. We also argue that simultaneously using different compounds to target parasites within mosquitoes and hosts alleviates selective pressure for resistance against drugs used exclusively to treat humans. Finally, we highlight critical knowledge gaps that must be filled to ensure that using antimalarial compounds to target parasites within mosquitoes is as robust as possible against parasite counter-evolution.

## Incorporating targeting of parasites during sporogony into epidemiology

Since the pioneering work of Ronald Ross in the early 20th century [22], mathematical modelling has played a pivotal role in malaria research [23]. In epidemiological applications, mathematical models are used to estimate the efficacy of malaria control measures on parasite transmission. The fitness of malaria parasites is often described by the basic reproductive number, or *R*_0_, which is defined as the number of secondary human infections caused by a single infected human [24]. Mathematically,

[1] $$R_{0}=\frac{m\;a^{2}b\;c\;e^{-gn}}{rg}$$

where *m* is the ratio of mosquitoes to humans, *a* is the rate at which humans are bitten, *b* is the infectivity, to human, of the parasites within mosquitoes, *c* is the infectivity of parasites within humans to mosquitoes, *g* is the mosquito mortality rate, *n* is the **extrinsic incubation period** (**EIP**), and *r* is the human recovery rate. The effects of different interventions on malaria transmission can be interpreted by examining their impacts on each of these model parameters and subsequently on *R*_0_. Assuming that a single infection is introduced to a fully naïve host population, *R*_0_ > 1 signifies that the infection will spread in the population. Conversely, the infection fails to spread when *R*_0_ ≤ 1.

**Figure 2.**
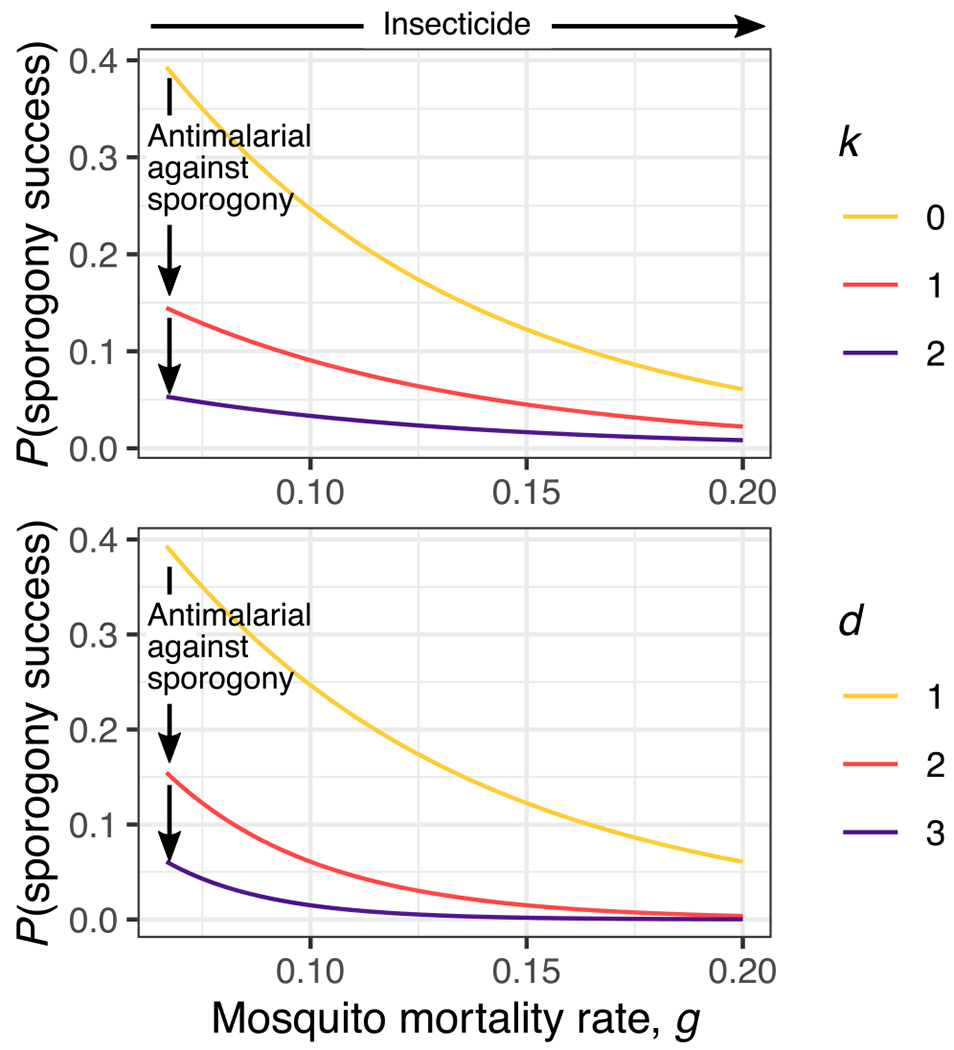
Sporogony-targeting compounds in mosquitoes that kill (top, *k*) or slow (bottom, *d*) parasite development coupled with insecticides that reduce adult mosquito lifespan (x axis) work synergistically to reduce the probability of successful sporogony, and hence onward transmission from exposed mosquitoes. For this illustration, the extrinsic incubation period, *n*, is set to 14 days. The daily mosquito mortality rate, *g*, is varied between 15^−1^ and 5^−1^ days (x axis) to demonstrate the impact of an insecticide that reduces mosquito lifespan. Nonetheless, exact numerical values of *n* and *g* do not qualitatively impact the synergistic benefit of combining a sporogony-targeting compound and an insecticide. In each panel, the yellow line (i.e., *k* = 0 and *d* = 1, respectively) represents the scenario where the insecticide is used without a sporogony-targeting compound.

The impact of targeting parasites during sporogony is mediated through the probability of a plasmodium-exposed mosquito eventually becoming infectious, and it is distinct from existing interventions (Figure 2). Mathematically, this probability is usually expressed as *e^−gn^* where *g* is the mosquito mortality rate and *n* is the EIP [24]. The phenotypic impact of a sporogony-targeting compound depends on the timing of exposure. Parasites exposed early in sporogony must survive the cytotoxicity of the compound, the probability of which can be expressed as *e^−k^* where *k* is the rate of parasite removal: for example, atovaquone exposure can effectively removes all parasites at the zygote-ookinete transition [21], indicating a very high *k* value. Exposure to atovaquone later in sporogony slows oocyst development, effectively prolonging the EIP by a proportion d. For example, Paton *et al.* observed a 45% decrease in oocyst developmental rate following tarsal exposure, that is, *d* = 1.45 [14]. Assuming a 10-day mean mosquito lifespan and 14-day EIP, a 45% delay in oocyst development corresponds to a nearly 50% reduction in the probability of completing sporogony (i.e., 13% compared to 25% in untreated mosquitoes). Combined, the probability of successful sporogony can be expressed as:

[2] $$e^{-\left(g\;n\;d\;+\;k\right)}$$

This simple equation highlights that a compound that kills ookinetes and/or slows oocyst development is effective alone, and that synergistic gains could be made by coupling it with an effective insecticide that reduces the mosquito’s lifespan (Figure 2). Scaling up these effects to the population level, an epidemiological model predicts that targeting sporogony is an effective approach to reduce malaria transmission [21]. Estimating the parameters *d* and *k*, and understanding how they are moderated in a real-world context – including by dose, timing, and frequency of exposure, genetic and environmental factors of parasite and vector – are key to predicting how effectively targeting parasites during sporogony will suppress malaria transmission.

## Evolutionary considerations for targeting parasites in mosquitoes

Parasite counter-evolution to resist or tolerate drugs is ubiquitous, if not inevitable [25]. The key questions are how likely is drug resistance/tolerance (i.e., how soon will it arise), and how rapidly will it spread? In this section, we evoke evolutionary theories to explore long-term consequences of chemically targeting sporogony (Box 1). We focus on likely differences in the *de novo* emergence and spread of resistance when parasites are targeted in mosquitoes versus humans, the evolutionary interests of parasites and mosquitoes, and the potential for alleviating selection on parasites to evade other malaria control measures.

### *De novo* emergence

Population size is a key determinant of ***de novo* emergence** of resistant mutants; the more individuals there are, the greater the potential source of a rare resistant mutant. Malaria parasites undergo extensive replication in the vertebrate host, first in the liver and then during the blood stage of infection. In the blood, each asexual-stage *P. falciparum* parasite produces 8–24 progeny in a cycle that repeats every 48 h [26], rapidly generating numbers that exceed 10^11^ [27]. Even at such high densities, spontaneous resistance mutation is expected to occur only in a single parasite at the peak of infection in a human [28]. Inside mosquitoes, sporogony involves only asexual replication during the oocyst phase, in which each oocyst (of which there are usually <10 per mosquito) produces around 10^3^ progeny [8,29]: thus, the peak parasite population within a mosquito is unlikely to exceed the number produced during the liver phase alone. Consequently, the far smaller number of genome replications during sporogony means fewer opportunities for *de novo* mutations to occur in the parasite’s genome within a mosquito than within a human.

Rare parasite mutants that do arise in mosquitoes may also be less likely than resistant counterparts within humans to be transmitted to the next phase in the life cycle. Specifically, mosquitoes are more likely to break infection chains due to their higher extrinsic mortality risk compared to humans, coupled with the EIP being long relative to the mosquito lifespan [30,31]. Failed onward transmission from mosquitoes may also be caused by a mosquito injecting sporozoites into an incompetent (e.g., livestock) or an immune human [32]. Thus, all else being equal, a resistant parasite mutant arising in a mosquito is less likely to be successfully transmitted to a human, than a resistant mutant arising in a human being transmitted to a mosquito.

Preventing the spread of antimalarial resistance/tolerance (that is naturally present in genetically diverse populations) is generally of higher priority than guarding against the emergence of *de novo* mutations conferring resistance. For example, resistance against sulfadoxine–pyrimethamine is driven by a single mutation that swept across the globe [33–35]. Similarly, resistance to chloroquine is linked to only a handful of independent spontaneous mutations [36]. These observations contrast with bacterial and viral infections where within-host *de novo* mutations are an eminent concern in every infection.

### Spread of resistance

In the absence of drug treatment, the fitness cost of a resistance mutation may select against its onward transmission, particularly in high-transmission settings where natural selection is more effective. For instance, *K13* gene mutations that grant partial resistance/tolerance against artemisinin – at the expense of slower replication – remain relatively rare in sub-Saharan Africa, where high levels of acquired immunity generates a large proportion of subclinical infections that are left untreated [37]. Drug use accelerates the spread of drug resistance because resistant parasites have a survival advantage over sensitive strains in drug-treated infections. Below, we outline two aspects of malaria and mosquito biology that may differentially shape the trajectory of the spread of drug resistance when humans versus mosquitoes are treated.

#### Duration of infection.

One adaptation to withstand drug treatment is the formation of slow-growing or growth-arrested cells that transiently enter a state of diminished metabolism and replication (often referred to as dormancy or quiescence) [38]. Temporary dormancy is taxonomically widespread (e.g., mammalian cancer cells [39], fungi [40], bacteria [41], *Trypanosoma* [42], *Toxoplasma* [43], *Leishmania* [44], *Plasmodium* [45]) and poses a pervasive concern for successful treatment against many diseases. Among malaria parasites, there are two known types of growth-arrested form. First, a small proportion of liver-stage parasites (known as hypnozoites) of *Plasmodium vivax* and *Plasmodium ovale* enter a growth-arrested stage, which can last for years and which are not vulnerable to most antimalarials [46]. Second, exposure to a variety of drugs (e.g., pyrimethamine [47], artemisinin [45], atovaquone [48], and difluoromethylornithine [49]) can induce dormancy in the blood stage of *P. falciparum* and other *Plasmodium* spp. that lasts for days to weeks, allowing parasites to survive periods of cytotoxic stress.

Human malaria infections can last for months to years. During this time, there are ample opportunities for parasites to enter a state of protective dormancy and recrudesce even after drugs with a long half-life have dissipated from the host. In contrast, parasites face a tight race against time during sporogony: in the field, adult female Anopheline mosquitoes live for less than 10 days on average [31], while *P. falciparum* requires between 1 and 2 weeks, depending on environmental temperature [30] and blood-feeding rates [50]. Only a portion of this already short lifespan is available for sporogony because females do not become ready to blood feed for up to 2 days after emerging as adults [51]. Also, several days may pass before a female can locate and successfully feed on a malaria-infected human, particularly in low-transmission areas. Thus, pressure to complete the EIP before the vector dies constrains the benefits of dormancy to wait out cytotoxic stress caused by chemical treatment. This constraint becomes more severe if insecticides that increase mosquito mortality are deployed alongside antimalarials targeting parasites during sporogony [21]. Thus, the inherently short duration of time available for sporogony is likely to be an asset for managing the spread of resistance.

#### Within-mosquito competition.

Malaria infections in high-transmission settings usually contain multiple genotypes [52]. Therefore, drug-resistant parasites typically share their human host with sensitive genotypes and compete for common finite resources (e.g., red blood cells) [53]. When genetically mixed infections remain untreated, resistant parasites are usually competitively inferior due to the metabolic cost of resistance. This competitive suppression of resistant genotypes slows the spread of drug resistance [54,55]. But, when the host is treated, resistant genotypes gain two benefits: first, they multiply faster than sensitive genotypes; second, they exploit the greater share of resources previously dominated by drug-sensitive competitors (i.e., competitive release). The greater the extent of suppression by competition in untreated infections, the greater the relative benefits of release from competition in drug-treated infections [25]. Consequently, a combination of wide drug coverage and a high prevalence of genetically diverse infections could facilitate the spread of drug resistance [54]. Epidemiological data from Angola, Ghana, and Tanzania support the role of within-host competition in accelerating the decline of chloroquine-resistant *P. falciparum* following the phase-out of chloroquine as the primary therapy [56].

To predict how within-mosquito competition shapes the spread of resistance against a sporogony-targeting compound, a fuller understanding of the frequency and extent of intraspecific competition is required. Infections involving multiple conspecifics are the rule rather than exceptions in human malaria infections [57]. Yet, whether the multiplicity of infections between humans and mosquitoes correlates closely does not have a consensus. Recent studies in a high-transmission setting report higher diversity in mosquitoes than in humans [58,59], but the opposite has also been observed [60]. On one hand, recombination and reassortment following mating during sporogony generate genetic diversity [61]. On the other hand, mosquitoes take a tiny amount of blood when feeding on a human, thus this ‘sample’ may not contain the full repertoire of parasite diversity within the human [60]. Furthermore, population bottlenecks (i.e., during the liver stage in humans, and mating and oocyst formation during sporogony) coupled with the potential for sequential acquisition of genotypes from multiple blood meals could generate complex dynamics in the multiplicity of infections in both humans and mosquitoes.

While competition between different malaria species in mosquitoes has been documented [62], the nature of intraspecific competition during sporogony remains an open question. Circumstantially, *P. falciparum* parasites found in the same mosquito tend to be closely related even in high-transmission areas where genetically diverse infections are expected a priori [63]. Such an observation is consistent with a wide range of competitive interactions: for example, rapid competitive exclusion of inferior strains or a strong priority effect that favours early invaders. However, facilitation may also occur in which already-infected mosquitoes are more permissive to subsequent infections [64]. Experimental manipulations using model systems can provide causal understanding of intraspecific interactions during sporogony and provide proof of principle for the roles that competitive suppression may play in constraining resistance to compounds targeting sporogony.

### Decoupling the evolutionary interests of parasites and their vector

Insecticides interrupt malaria transmission by shortening the mosquito’s lifespan. Thus, as mosquitoes evolve resistance against an insecticide, the loss of transmission is mitigated through mosquito evolution, potentially at no cost to the parasites themselves [5]. Some compounds that can target sporogony also have adverse fitness consequences for mosquitoes. For example, the pyrocatechol RC-12 damages the mosquito’s salivary glands [65]. Unfortunately, a compound that simultaneously affects mosquito and parasite fitness selects for mosquito and parasite counter-evolution simultaneously, and adaptation by only one party would render the compound less effective at supressing transmission.

Conversely, if a sporogony-targeting compound does not affect mosquito fitness, no selection is imposed on mosquitoes. Thus, parasites rely solely on their own gene pool and genetic machinery to find an evolutionary solution. One such compound is atovaquone, which does not impact mosquito fitness as measured by fecundity and lifespan, at least under laboratory conditions [14,21]. Thus, it is possible to target parasites within mosquitoes in a manner that decouples the evolutionary interests of parasites and mosquitoes. A priori, facing one instead of two evolutionary foes simultaneously is a favourable evolutionary gambit for an intervention.

### Shifting selection pressure away from existing control tools

Every major class of antimalarial compounds has been tested for efficacy against parasites in sporogony [7,20]. However, we do not advocate using current antimalarials against mosquito-stage parasites. Using chemical compounds with shared pathways of action in multiple contexts could conceivably exacerbate selection for drug resistance. Instead, just as combination therapy involves complementary drugs with distinct action pathways [6], chemicals with independent modes of action should be used to treat humans and deployed against parasites during sporogony.

Using dedicated compounds to target parasites during sporogony offers an additional – rather than an alternative – means of control, which could alleviate the task of suppressing transmission away from human antimalarials. A **radical cure** is difficult to achieve with antimalarials targeting asexual blood-stage parasites, yet it is necessary to prevent transmission. Instead, sporogony-targeting compounds can take the burden of transmission suppression away from (other) antimalarials used to treat humans. This allows antimalarials to be used in humans with the sole goal of mitigating symptoms (instead of aiming for clearance), enabling lower doses to achieve maximal clinical benefit with minimal side effects [25]. Furthermore, by reducing the dose and duration of human antimalarial regimes, the spread of resistance can be slowed and even reversed. For example, chloroquine resistance was widespread but declined in Malawi [66], Kenya [67], and Tanzania [68] following a withdrawal of chloroquine in favour of artemisinin-based combination therapy. Radical cure may be replaced in the future by a more effective combination of separate exposure to different compounds within humans and mosquitoes.

## Concluding remarks

A sound drug stewardship programme is required to alleviate the risk of the emergence of drug resistance, slow its spread, and maximise the drug’s lifespan. Targeting parasites during transmission via mosquitoes adds an epidemiologically novel mode of malaria control untapped by existing intervention tools. We outline favourable evolutionary considerations specific to chemically targeting parasites during sporogony, including relieving some pressure on the pharmaceutical treadmill for antimalarials to specifically treat humans. Several key questions need to be addressed before quantitative predictions for the evolutionary consequences of chemically targeting parasites during sporogony can be made (see Outstanding questions). These questions reflect general knowledge gaps in within-vector parasite ecology, the pharmacokinetics and pharmacodynamics of sporogony targeting compounds inside mosquitoes and how these depend on the deployment approach, and practicalities and economics of deployment options, which must be addressed going forward.

## Box 1. Anticipating and monitoring evolutionary changes

We recommend establishing protocols to monitor the spread of resistance at the population level before sporogony-targeting compounds are deployed in the field. First, extensive screening of the **standing genetic variation** is desired because genetic variation fuels evolutionary changes. In the rodent malaria parasite *Plasmodium chabaudi*, there is considerable standing variation in artemisinin sensitivity at the blood stage [69]. If less-susceptible variants exist before a sporogony-targeting compound is deployed, the spread of resistance does not rely on new variants arising through *de novo* mutations. High standing variation may also reflect low metabolic cost of resistance because these variants would be poor competitors and selected against otherwise. Screening for variants is also useful because the genetic makeup of pre-existing low-susceptibility variants can inform the development of resistance markers.

Second, resistance markers can also be identified by rearing parasites over multiple generations under pressure from each sporogony-targeting compound to select for resistance evolution in a laboratory setting [55]. Conducting such experiments with the full life cycle in each selection cycle is extremely challenging, but results from the responses of selection on blood stages, which are more tractable, might provide limited insight due to different metabolic pathways used by blood and sporogony stages.

Third, resistance monitoring of blood-stage parasites has long relied on phenotypic assays (e.g., clearance curves [70]). A recent development advocates simultaneous measurements of the *in vivo* drug concentration and parasite density to determine the dose–response relationship in parasite recrudescence and reinfection [71]. Such an assay identifies potentially resistant infections without genetic markers and provides early warning signals for resistance [71]. Similarly, *in vivo* compound concentrations and parasite phenotypes at different stages of sporogony should be assessed to monitor changes in susceptibility and distinguish resistance/tolerance from inadequate dose exposure in mosquitoes. Relevant *in vivo* phenotypic markers already exist for sporogony, (e.g., ookinete killing and oocyst developmental rate [14,21]) but monitoring pharmacokinetics and pharmacodynamics within mosquitoes is likely to be challenging. Once identified, simple mathematical formulations (e.g., *R*_0_ and vectorial capacity) can be used to estimate the epidemiological consequence of phenotypic variation (of parasites and/or mosquitoes) at the population level.

Fourth, estimating the risk of *de novo* resistance emergence against compounds targeting parasites during sporogony will require a mathematical model that explicitly tracks parasite development from the ingestion of a bloodmeal to onward transmission to humans, and all stages in between. Although the population dynamics of malaria parasites within mammalian hosts have been studied extensively (e.g., [71–74]), mechanistic modelling of within-mosquito parasite dynamics is a relatively recent development (e.g., [31,62,75–77]). Future studies should extend these models to provide a quantitative understanding of the origin and fate of resistance mutations in mosquitoes. A key advantage of a mechanistic model lies in its flexibility to explore outcomes across contexts, for example, assessing the impacts of temperature which strongly modulates the course of sporogony.

Finally, the impacts of antimalarials on sporogony include partial to complete parasite removal and developmental delay (see Figure 1 in main text), suggesting that tolerance and resistance could occur through quantitative changes, rather than a binary switch between susceptible and resistant genotypes. Modelling frameworks rooted in evolutionary game theory [77] and quantitative genetics [78] – which have been developed to study the evolution of quantitative parasite traits (e.g., virulence) – will provide useful tools for exploring the long-term evolutionary consequences of targeting parasites inside mosquitoes.

## Outstanding questions

Possibility of effective radical cure. Is full clearance of parasites during sporogony likely? While radical cure is the conventional wisdom, complete clearance of the within-human parasite population is difficult at the blood stage. To determine whether radical cure is possible, detailed knowledge of within-mosquito compound pharmacokinetics, pharmacodynamics, and properties in the environment (e.g., chemical half-life on bed nets and in sugar bait) is required. If radical cure is unlikely, does a vast reduction in sporozoite number effectively curtail onward transmission?

Residual drug concentration. Do chemicals used to target sporogony transfer to humans upon subsequent blood feeding? If mosquitoes pass on residual chemicals to humans, toxicological consequences of such secondary exposure need to be considered. Can residual compounds in the human host continue to select for resistance against the compound?

Standing variation in drug sensitivity. Is there tolerance to compounds that can target sporogony in the contemporary gene pool of malaria parasites?

Within-mosquito parasite competition. How do conspecific genotypes interact within the mosquito, and does the nature of interactions vary between the different stages of sporogony? Is there competition, and so, the potential for competitive release?

Drug coverage. The selection pressure for resistance scales with the proportion of parasites exposed to the drug. How does the proportion of infected mosquitoes exposed to compounds that target sporogony compare with coverage arising from human treatment?

Cost of resistance across mosquito and human stages. If parasites evolve tolerance/resistance against sporogony-targeting compounds, does this involve fitness costs that manifest in the human phase of the life cycle?

## Highlights

Counter-evolution of the targeted organisms (i.e., mosquitoes resist insecticides, and parasites resist antimalarial drugs) threatens the future of malaria control.

The discovery of new compounds for either insecticides or antimalarials is being outpaced by evolution, and so novel interventions to augment the current arsenal are urgently needed.

A promising new approach is to target malaria parasites inside mosquitoes (i.e., sporogony) which is considered one of the most vulnerable stages of the parasite’s entire malaria life cycle. Many chemical compounds have been shown to be effective against *Plasmodium* parasites during sporogony.

Deploying a new control tool is ideally undertaken within a stewardship programme that minimises the risk of resistance evolution and slowing its spread. Yet, evolutionary consequences of targeting malaria parasites in mosquitoes remain unexplored.

## Glossary

*de novo* emergence: the appearance of a particular genotype or phenotype for the first time in a population.
Drug resistance/tolerance: resistance refers to parasite genetic adaptation that reduces the effectiveness of a drug used to suppress parasites. Partial resistance is often referred to as tolerance. Tolerance may result from pre-existing parasite traits/behaviours that reduce drug efficacy.
Extrinsic incubation period (EIP): the duration of parasite development in an insect vector from the point of ingestion in a blood meal until parasites are ready to be transmitted from the salivary glands to a new host. The EIP is also referred to as the ‘duration of sporogony’.
Gametocyte: the transmission stage of malaria parasites produced during the blood-stage infection and ingested by an insect vector upon blood feeding.
Oocyst: a parasite cyst attached to the basal layer of the mosquito’s midgut; it incubates sporozoites.
Ookinete: the malaria parasite’s motile zygote that penetrates the mosquito’s midgut.
Radical cure: aggressive drug treatment that aims to remove the entire pathogen/parasite population from a patient. If the treated host already contains a drug-resistant/tolerant variant, aggressive chemotherapy necessarily grants a strong selective advantage to parasites that are even slightly less susceptible than the rest.
Sporogony: the process of parasite development in mosquitoes that involves sexual reproduction and migration through several mosquito organs and tissues. The beginning is marked by the ingestion of gametocytes in a blood meal. Sporogony concludes when sporozoites invade the mosquito’s salivary glands, ready for onward transmission to a mammalian host.
Sporozoite: a motile stage of malaria parasites that invades the mosquito’s salivary glands and is responsible for transmission to mammalian hosts.
Standing genetic variation: diversity of alternative genotypes simultaneously present in a population.
